# Structural Characterization and Comparison of Monovalent Cation-Exchanged Zeolite-W

**DOI:** 10.3390/ma13173684

**Published:** 2020-08-20

**Authors:** Donghoon Seoung, Hyeonsu Kim, Pyosang Kim, Chihyun Song, Suhyeong Lee, Sungmin Chae, Sihyun Lee, Hyunseung Lee, Yongmoon Lee

**Affiliations:** 1 Department of Earth Systems and Environmental Sciences, Chonnam National University, Gwangju 61186, Korea; dseoung@jnu.ac.kr (D.S.); 197942@jnu.ac.kr (H.K.); 197944@jnu.ac.kr (P.K.); 2 Department of Geological Sciences, Pusan National University, Busan 46241, Korea; autumnleaves@pusan.ac.kr (C.S.); norium@pusan.ac.kr (S.L.); sjk04107@pusan.ac.kr (S.C.); tlgus745@pusan.ac.kr (S.L.); hslee07@pusan.ac.kr (H.L.)

**Keywords:** zeolite-W, cation form, synchrotron X-ray diffraction, Rietveld refinement

## Abstract

We report comparative structural changes of potassium-contained zeolite-W (K-MER, structural analogue of natural zeolite merlinoite) and monovalent extra-framework cation (EFC)-exchanged M-MERs (M = Li^+^, Na^+^, Ag^+^, and Rb^+^). High-resolution synchrotron X-ray powder diffraction study precisely determines that crystal symmetry of MERs is tetragonal (*I4/mmm*). Rietveld refinement results reveal that frameworks of all MERs are geometrically composed of disordered Al/Si tetrahedra, bridged by linkage oxygen atoms. We observe a structural relationship between a group of Li-, Na-, and Ag-MER and the group of K- and Rb-MER by EFC radius and position of M(1) site inside double 8-membered ring unit (*d8r*). In the former group, *a*-axes decrease reciprocally, *c*-axes gradually extend by EFC size, and M(1) cations are located at the middle of the *d8r*. In the latter group, *a*- and *c*-axes lengths become longer and shorter, respectively, than axes of the former group, and these axial changes come from middle-to-edge migration of M(1) cations inside the *d8r* channel. Unit cell volumes of the Na-, Ag-, and K-MER are ca. 2005 Å^3^, and the volume expansion in the MER series is limited by EFC size, the number of water molecules, and the distribution of extra-framework species inside the MER channel. EFC sites of M(1) and M(2) show disordered and ordered distribution in the former group, and all EFC sites change to disordered distribution after migration of the M(1) site in the latter group. The amount of water molecules and porosities are inversely proportional to EFC size due to the limitation of volume expansion of MERs. The channel opening area of a *pau* composite building unit and the amount of water molecules are universally related as a function of cation size because water molecules are mainly distributed inside a *pau* channel.

## 1. Introduction

Porous materials have been acknowledged as important specimens due to their pore characteristics, which are dependent on pore morphology, pore size, and pore size distribution [1,2,3]. Numerous experiments have been conducted to the understand fundamental factors of porous materials, and some have suggested characterization methods, e.g., the work of Liu et al. (2014) [4]. Zeolites are crystalline and are one of the popular functional materials that have been widely studied under various pressure, temperature, and chemical composition conditions for several decades [5,6,7,8,9,10]. Small-pore zeolites have been utilized in research areas for applications such as water purification, H-storage media, NO_x_ membrane, and CO_2_ removal due to their accessible size, 8-membered ring composition, and selective catalytic reaction [11,12,13,14]. Zeolite-W is a synthetic phase whose framework topology is the same as the natural small-pore zeolite, merlinoite (assigned zeolite code: MER) [15,16]. The aspect of crystal structure for zeolite-W is usually determined as a tetragonal (*I4/mmm*) or orthorhombic (*Immm*) space group. Its framework is composed of a secondary building unit (SBU) of Si/Al tetrahedra such as 4-membered rings (4MR), 8-membered rings (8MR), and double 8-membered rings. A channel system usually consists of three-dimensional connections of 8MR pores [16]. Zeolite-W typically has an intermediate Si/Al framework ratio of 2 < Si/Al ≤ 5 and has been synthesized through several different routes, such as in the system of Na_2_-K_2_O-SiO_2_-Al_2_O_3_, for controlling Si/Al ratio and pore size [17,18,19]. Many studies have provided information as to synthesis, crystal growth, and physiochemical characterization using methods such as Fourier transform infrared spectroscopy, thermogravimetric analysis, and solid-state NMR; however, there are few reports about the crystal structure of prepared products [19,20,21,22]. X-ray diffraction is one of the basic methods used to characterize products in order to understand heterogenetic cation sites, framework topology, and distributions of zeolitic water inside channels.

We have successfully prepared zeolite-W (K-MER) and its fully exchanged monovalent cation forms (Li-, Na-, and Ag-MER) by using conventional hydrothermal synthesis, following the work of Itabashi et al., and the ion-exchange method, respectively [23]. Here, we report comparative and systematic changes in crystal structures and chemical composition of a monovalent M-MER series at ambient conditions using high-resolution synchrotron X-ray powder diffraction, Rietveld structure refinement, energy dispersive X-ray spectroscopy (EDS), and thermogravimetric analysis (TGA).

## 2. Materials and Methods

### 2.1. Sample Preparation

The starting material, zeolite-W (K-MER), was synthesized under hydrothermal conditions according to Itabashi et al. [23]. An amount of 50 wt % of aqueous solution of KOH (Sigma-Aldrich) and Al(OH)_3_ (Sumitomo Chemical Co. Ltd., Tokyo, Japan) was mixed and heated on a hot plate to prepare a potassium aluminate solution. Colloidal silica (Sigma-Aldrich) and a calculated amount of water were then added to the potassium aluminate solution. The batch composition was similar to 3 K_2_O:1.5 Al_2_O_3_:5 SiO_2_:100 H_2_O. For crystallization, the mixture was put into autoclave and then heated at 150 °C for 48 h. The final product was washed and dried in ambient conditions. Other cation forms were prepared by fully saturated MCl solution (M = Li^+^, Na^+^, and Ag^+^) and K-MER powder in a 100:1 ratio of solution volume to powder weight. The mixture was stirred at 80 °C in a closed system, minimizing the loss of water. After 24 h, the solid was separated from the solution by vacuum filtration. The dried powder was then subjected to two more exchange cycles. The final product was washed with a deionized water and subsequently air-dried at ambient conditions. Stoichiometric analyses were performed on products using EDS, and we confirmed that over 99% of the cation was exchanged. To determine the amount of H_2_O molecules, TGA was used with a heating range of 25 to 800 °C and a heating rate of 10 °C/min under a nitrogen atmosphere (Figure S3). Experimental conditions for cation exchange and chemical analysis results are summarized in Tables S1 and S2.

### 2.2. Synchrotron X-ray Powder Diffraction

High-resolution synchrotron X-ray powder diffraction data were collected for precise determination of crystal symmetry of MERs at the 9B beam line of Pohang Light Source-II (PLS-II) at the Pohang Accelerator Laboratory (PAL). The incident X-rays were vertically collimated by a mirror, and they were monochromatized to a wavelength of 1.4865(1) Å using a double-crystal Si (111) monochromator. The detector arm of the vertical scan diffractometer was composed of six sets of Soller slits, flat Ge (111) crystal analyzers, antiscatter baffles, and scintillation detectors, with each set separated by 21° in 2-theta. Each specimen of approximately 0.2 g powder was prepared using a flat plate side loading method. Step scans were performed at room temperature from 8° in 2-theta with 0.005° increments and 2° overlaps of the detector bank, up to 128.5° in 2-theta. Synchrotron X-ray powder diffraction data were measured in a transmission geometry to avoid preferred orientation at the 3D and 5A beam lines of Pohang Light Source-II (PLS-II) at the Pohang Accelerator Laboratory (PAL) in South Korea. The primary white beam from the bending magnet of 3D or the superconducting insertion device of 5A was directed on a Si (111) crystal, and sets of parallel slits were used to create monochromatic X-rays with wavelengths of 0.6926(1) Å and 0.6199(1) Å for 3D and 5A, respectively. Each sample was loaded into a 0.5 mm diameter SiO_2_ glass capillary, and the capillary was subsequently sealed to minimize dehydration by atmosphere during measurement. All samples were measured with sample rotation for 5 min using the MAR345 image plate two-dimensional X-ray detector. The calibrations of the X-ray wavelength and sample-to-detector distance were achieved using a LaB_6_ standard (SRM 660c).

### 2.3. Structural Analysis by Rietveld Refinement

Unit cell length and volume changes were derived from a series of whole-profile fitting procedures using the General Structure Analysis System (GSAS) suite of programs [24]. The background was fixed at selected points, and a pseudo-Voigt profile function proposed by Thompson et al. was used to model the observed Bragg peaks [25]. The structural models of MERs were obtained by Rietveld refinements [24,26,27]. A March–Dollase function was used to account for preferred orientation [28]. In order to reduce the number of parameters, isotropic displacement factors were refined by grouping the framework species and the extra-framework species. Geometrical soft-restraints on the T–O (T = Si, Al) and O–O bond distances of the disordered Si/Al tetrahedra were applied by stoichiometric results of each sample from EDS: the distances of Si–O were restrained to values from 1.642 to 1.648 with esd of ±0.001 Å, and the O–O distances ranged from 2.692 to 2.688 with esd of ±0.005 Å. In the final stages of the refinements, the weights of the soft restraints were gradually reduced. This did not lead to any significant changes in the interatomic distances, and convergence was achieved by simultaneously refining scale factors, lattice constants, 2-theta zero, preferred orientation function, and the atomic positional and thermal displacement parameters. The final refined parameters are summarized in Table S3, and the selected bond distances and angles are listed in Table S4.

## 3. Results and Discussions

**Figure 1:**
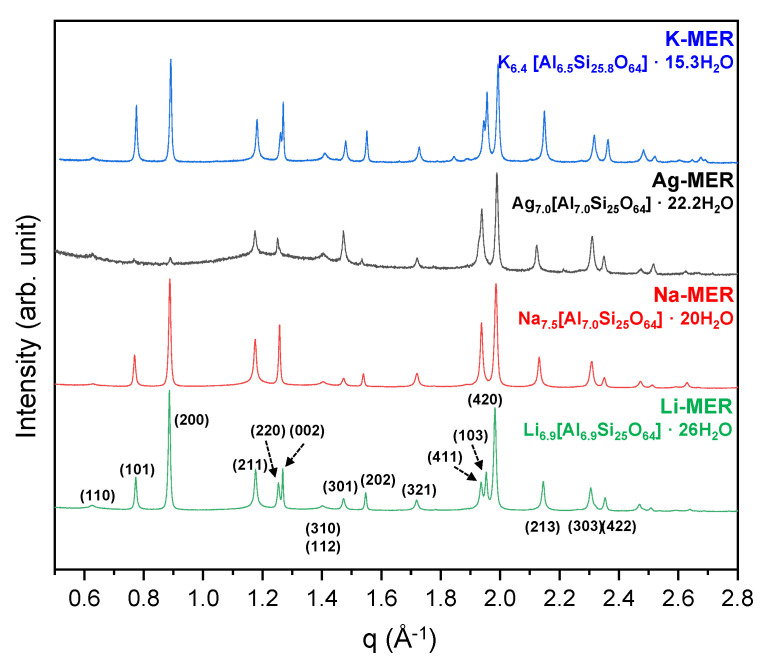
Changes in the synchrotron X-ray powder diffraction patterns of Li-, Na-, Ag-, and K-MER at ambient conditions. Miller indices are shown for the Bragg peaks. Chemical compositions are derived from our refinement results.

As described in the experimental section, we have successfully prepared K-contained zeolite-W and its series of monovalent cation-exchanged forms by Li^+^, Na^+^, and Ag^+^ (Li-, Na-, and Ag-MER, respectively). High-resolution synchrotron X-ray diffraction patterns of MERs at ambient conditions are shown in Figure 1. Chemical compositions derived from Rietveld refinement are Li_6.9_Al_6.9_Si_25.0_O_64.0_·26H_2_O, Na_7.5_Al_7.0_Si_25.0_O_64.0_·20.0H_2_O, Ag_7.0_Al_7.0_Si_25.0_O_64.0_·22.2H_2_O, and K_6.42_Al_6.5_Si_25.8_O_64.0_·15.3H_2_O for the Li-, Na-, Ag-, and K-MER, respectively (Table S1). Bragg peaks of each phase are indexed to crystal symmetry of a tetragonal system and space group *I4/mmm*. The peak positions such as (101), (200), (220), and (103), related to the *a*- and *c*-axis, in each MER phase seem slightly shifted in 2-theta, while relative peak intensities are obviously changed due to changes in chemical composition. It is therefore shown that distribution and occupancy changes of extra-framework species such as EFCs and water molecules, related to intensity changes, primarily arise from unit cell length changes, related to peak position changes by chemical composition.

**Figure 2:**
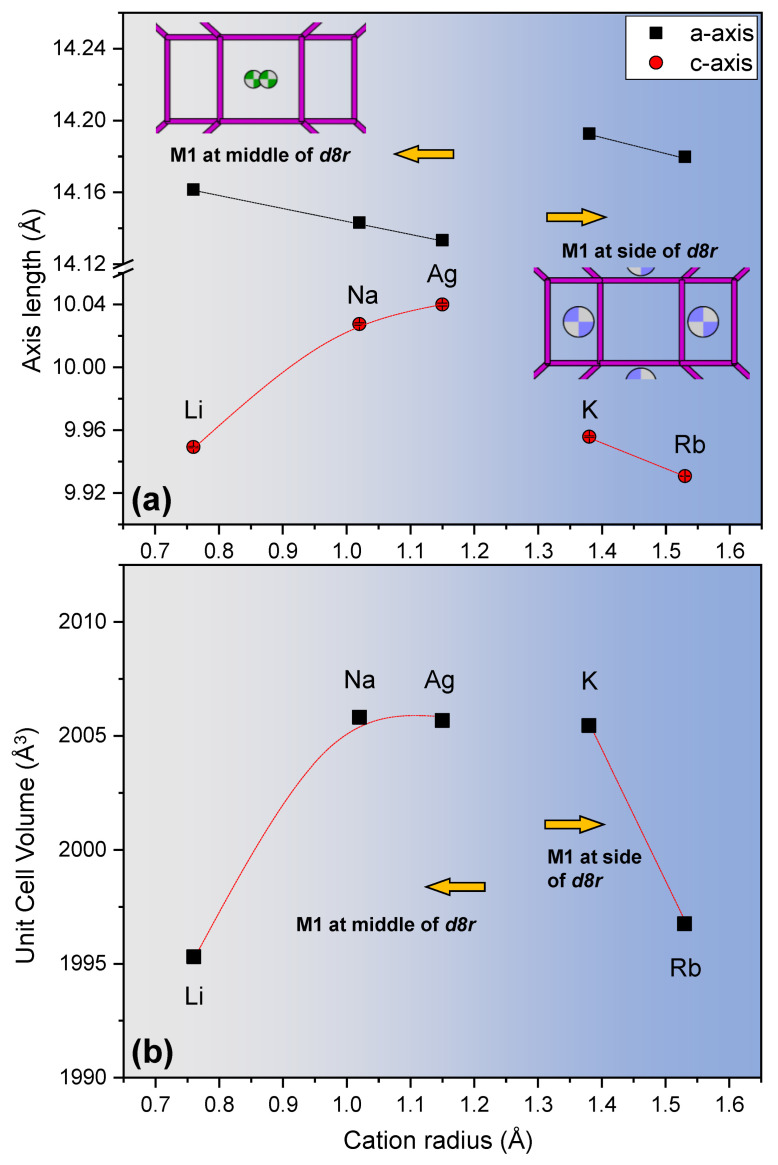
Refined (**a**) *a*- and *c*-axis and (**b**) volume of cation forms of MER as a function of extra-framework cation size.

Whole-profile fitting analysis reveals that unit cell parameters have two distinguishable relationships in the group containing the Li-, Na-, and Ag-MER and in the group containing the K- and Rb-MER (model from Itabashi et al.) by means of exchanged cation radius and location of M(1) site of each EFC (Figure 2). In the former group (Li-, Na-, and Ag-MER), the *a*-axes linearly decrease from 14.1613(4) to 14.1334(9) Å, and the M(1) (radius < 1.2 Å) site is located at middle of the *d8r* along (010) direction. EFCs of M(1) progressively migrate toward the side wall of the *d8r* as a function of EFC radius (atomic coordinate changes of *x* in Table S3). The bond valence sums (BVS) calculations of MERs show that M(1) cations usually have lower values than M(2) cations, which indicates M(1) cations are under-bonded in comparison with M(2) cations (Table S4) [29]. This helps to understand the reason why M(1) cations migrate easily in the *d8r* channel. The location of the M(1) site is abruptly changed to the side wall of *d8r* when cation radius is larger than the 1.35 Å of K^+^, which reflects abrupt increments of *a*-axes in the latter group (K- and Rb-MER). Similarly, the *a*-axes of this group linearly decrease from 14.1927(4) to 14.1798(3) Å by means of EFC size. In the case of *c*-axes lengths, they gradually extend up to 10.040(1) Å of the Ag-MER in the former group; meanwhile, *a*-axes decrease reciprocally by EFC size. The *c*-axis also suddenly collapsed before and after cation radius of 1.35 Å and then linearly decreased identically in the latter group (Figure 2a). Unit cell volume changes due to complex distribution and migration of the EFCs following their radius are shown in Figure 2b. Volumetric changes of M-MERs show gradual expansions and contractions, proportionally for Li^+^, Na^+^, and Ag^+^ and reciprocally for K^+^ and Rb^+^, as a function of cation radius. Interestingly, volumes of the Na-, Ag-, and K-MER are similar, ca. 2005 Å^3^, and they constitute ca. 5% volume expansion compared with the volume of Li-MER, ca. 1995.3(1) Å^3^. Thus, this indicates that the volume expansion in the MER series is limited to ca. 2005 Å^3^ through alkaline metal cation substitution.

**Figure 3:**
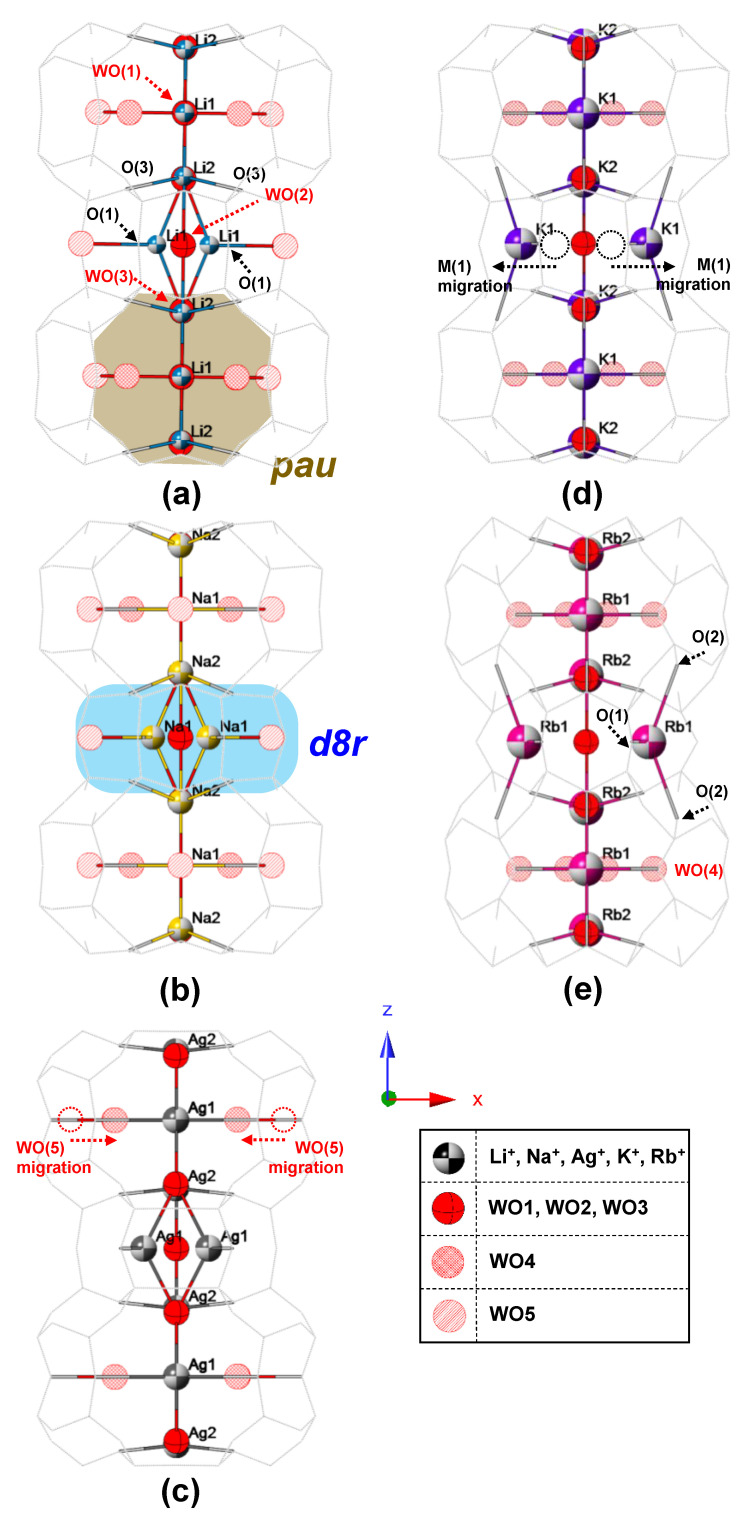
Polyhedral representations of (**a**) Li-MER, (**b**) Na-MER, (**c**) Ag-MER, (**d**) K-MER, and (**e**) Rb-MER along the (010) direction. The grey stick represents disordered Al/Si framework. Each colored beach ball represents an extra-framework cation. Equatorial, hatched, and striped red balls represent oxygens of WO(1)–WO(3), WO(4), and WO(5), respectively.

**Figure 4:**
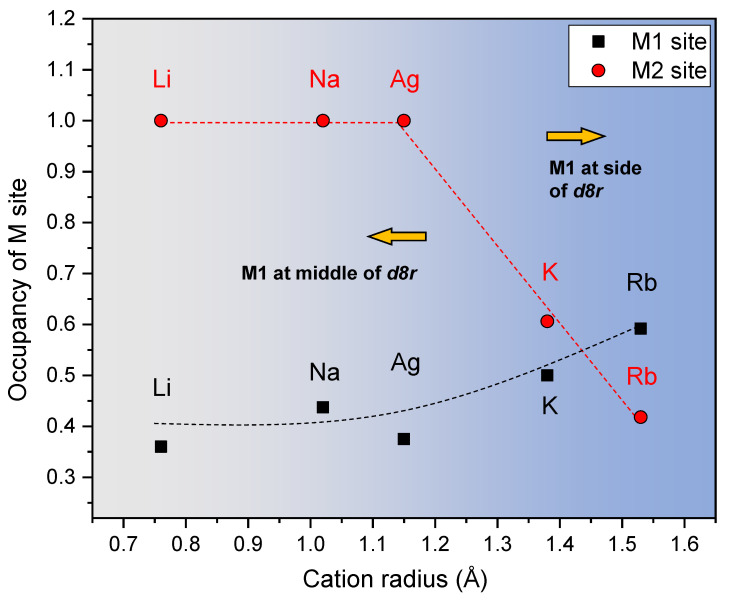
Changes of occupancies of M(1) and M(2) sites (M = Li^+^, Na^+^, Ag^+^, K^+^, Rb^+^).

Refined crystal structures of MERs are shown in Figure 3 and Figure S1. The EFC site of M(1) (colored beach balls) in the Li-, Na-, and Ag-MER is located at the middle of the *d8r* channel projected to the (010) direction (filled by light-blue color) and has two bonds with one framework oxygen, O(1), and one water molecule, WO(3), whereas the M(1) is near the edge of *d8r* and has three bonds with O(1), O(2), and WO(4) in the K- and Rb-MER. On the other hand, the EFC sites, M(2), are constantly located at the center of the channel along the *c*-axis despite cation changes. Water molecules (red symbols) are designated by five different sites, from WO(1) to WO(5). Sites of WO(1), (2) and (3) (equatorial balls) are located at the middle of the *pau* channel (filled by brown color) and *d8r* along the c-axis, regularly. Both WO(1) and (2) are connected with M(2) cations, and WO(3) is bonded with M(1) cations along the *c*-axis due to a different *z*-coordinate of WO(3) compared to that of WO(1) and (2). The sites of WO(4) and (5) (hatched and striped balls, respectively) are distributed around the edge of the *pau* channel and have one or two coordinates bonding with M(1) or themselves. Thus, the WO(4) and (5) easily migrate inside the channel, and sites are finally merged to WO(4) during migration of a larger cation, such as K^+^ and Rb^+^ in the K- and Rb-MER. Changes of occupancies of M(1) and (2) sites are related to cation size (Figure 4). In case of the Li-, Na, and Ag-MER, the M(1) and M(2) sites are partially (less than ca. 0.45) and fully occupied, respectively. As the M(1) cation migrates toward the side of *d8r* due to its size, the occupancy of M(1) gradually increases. The occupancies of M(2) are reduced to less than ca. 0.6 when cation radius is greater than 1.35 Å, in the case of the K- and Rb-MER, and the occupancy of M(1) increases more than that of M(2) in the case of the Rb-MER. These changes of occupancies are related to the number of coordinated bonds with framework oxygens. The M(1) cation in the Li-, Na-, and Ag-MER is connected to one framework oxygen, O(1), with bond distance of ca. 3–3.3 Å. The M(1) site becomes more stable by bonding with two framework oxygens, O(1) and (2), after its migration. From K- to Rb-MER, the M(1) cation is more tightly connected with O(1) than M(2)–O(3) bonding, and therefore occupancy of M(1), in the case of the Rb-MER, can further increase more than the K-MER.

**Figure 5:**
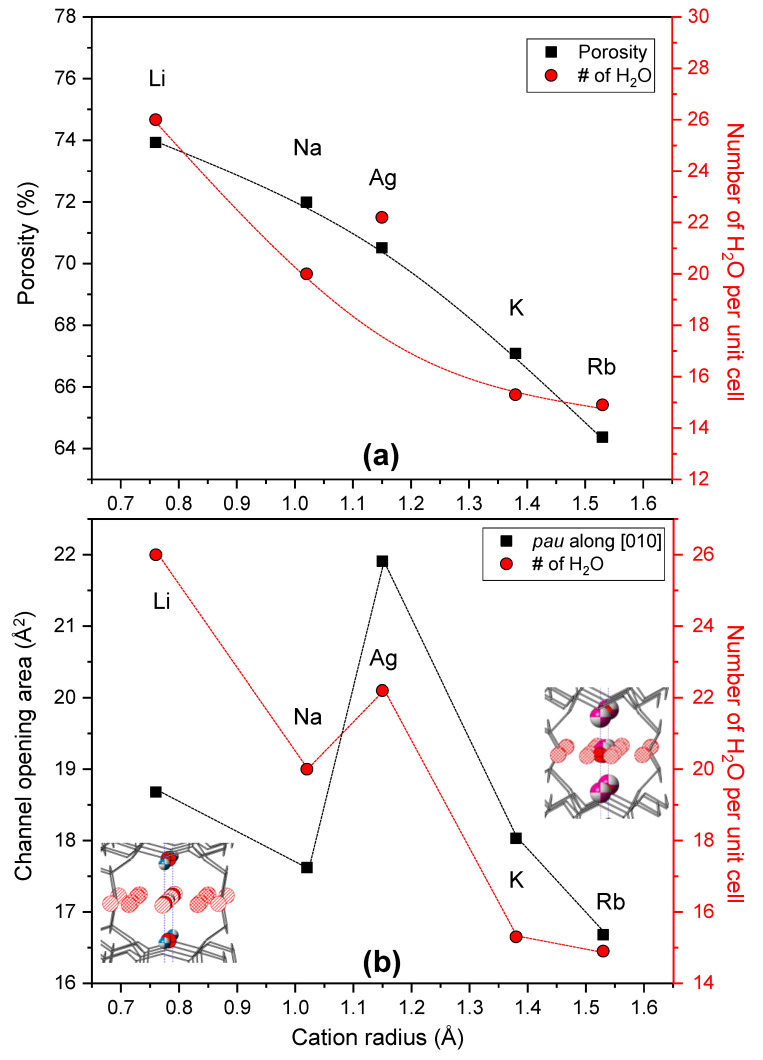
Changes of (**a**) porosity and amount of water molecules and (**b**) channel opening area of the *pau* unit and amount of water molecules as a function of extra-framework cation size.

We find universal relationships between porosity, channel opening area, and the number of water molecules in the unit cell as a function of cation radius (Figure 5). The porosity of all MERs is calculated from our structure models. The porosity and the number of water molecules are gradually and exponentially decreased by EFC size, respectively. Considering the limitation of the largest volume expansion among MER structures, we expect that the EFC size mainly influences a determination that the porosities and rearrangements of EFCs in the framework are accompanied by cation size in order to absorb water molecules as much as possible. In case of the Ag-MER, the value of water molecules per unit cell is greater than expected value on the trend line (red dotted line in Figure 5a), which might be due to the high electronegativity of the silver cation compared to other cations. The degree of channel opening area, along the (010) direction, of the *pau* composite unit is related to the number of water molecules. In our structure models, all water molecules are distributed inside a *pau* channel along *a*- or *b*-axis; therefore, changes of channel opening area are concomitantly observed in agreement with the number of water molecules along the *a*- or *b*-axis rather than the *c*-axis (Figure 5b and Figure S2).

## 4. Conclusions

In this work, we have demonstrated comparative crystal chemistry among synthesized K-MER and its monovalent cation forms of Li^+^, Na^+^, Ag^+^, and Rb^+^, along with several correlations by means of EFC size. The *a*- and *c*-axis show distinguishable changes between the Li-, Na-, and Ag-MER group and the K- and Rb-MER group depending on whether the cation site of M(1) is located at the middle or the side wall of the *d8r*. The maximal volume expansion is ca. 2005 Å^3^, and therefore porosity and the amount of water molecules inside the channel decrease with EFC size, accompanying migration of extra-framework species such as EFCs and water molecules. We expect that our results can provide fundamental knowledge to understand the relationship between crystal structure and chemical changes of MER-type zeolites, which can be applied to the enhancement of catalysts and absorbents.

## Supplementary Materials

The following are available online at https://www.mdpi.com/1996-1944/13/17/3684/s1, Figure S1: Polyhedral representations of (**a**) Li-MER, (**b**) Na-MER, (**c**) Ag-MER, (**d**) K-MER and (**e**) Rb-MER along (001) direction. Grey stick represents disordered Al/Si framework. Each colored beach ball represents extra-framework cation. Equatorial, hatched, and striped red balls represent oxygens of WO(1)–WO(3), WO(4), and WO(5), respectively, Figure S2: Comparison of channel opening area of *d8r* (black symbol) and *pau* (red symbol) along (001) direction and sum of two areas., Figure S3: Thermogravimetric analysis results of Li-, Na-, Ag-, and K-MER., Table S1: Cation-exchange conditions of MERs and chemical composition from Rietveld refinement and stoichiometric analysis., Table S2. Chemical composition calculated from Energy Dispersive Spectroscopy (EDS) method., Table S3: Refined cell parameters and atomic coordinates of M-MER at ambient condition (M = Li^+^, Na^+^, Ag^+^, K^+^ and Rb^+^)., Table S4: Selected interatomic distances (Å) and angles (°) for M-MER at ambient condition (M = Li^+^, Na^+^, Ag^+^, K^+^, and Rb^+^).
