# Supplementary material for: Structural Characterization and Comparison of Monovalent Cation-Exchanged Zeolite-W
Source: Materials (Basel). 2020 Aug 20;13(17):3684. doi: 10.3390/ma13173684 (PMC7503765; doi:10.3390/ma13173684)
Supplement: Supplementary file 1 [file materials-13-03684-s001.pdf]

materials

MDPI

Supplementary data

Structural Characterization and Comparison of Monovalent Cation-Exchanged Zeolite-W

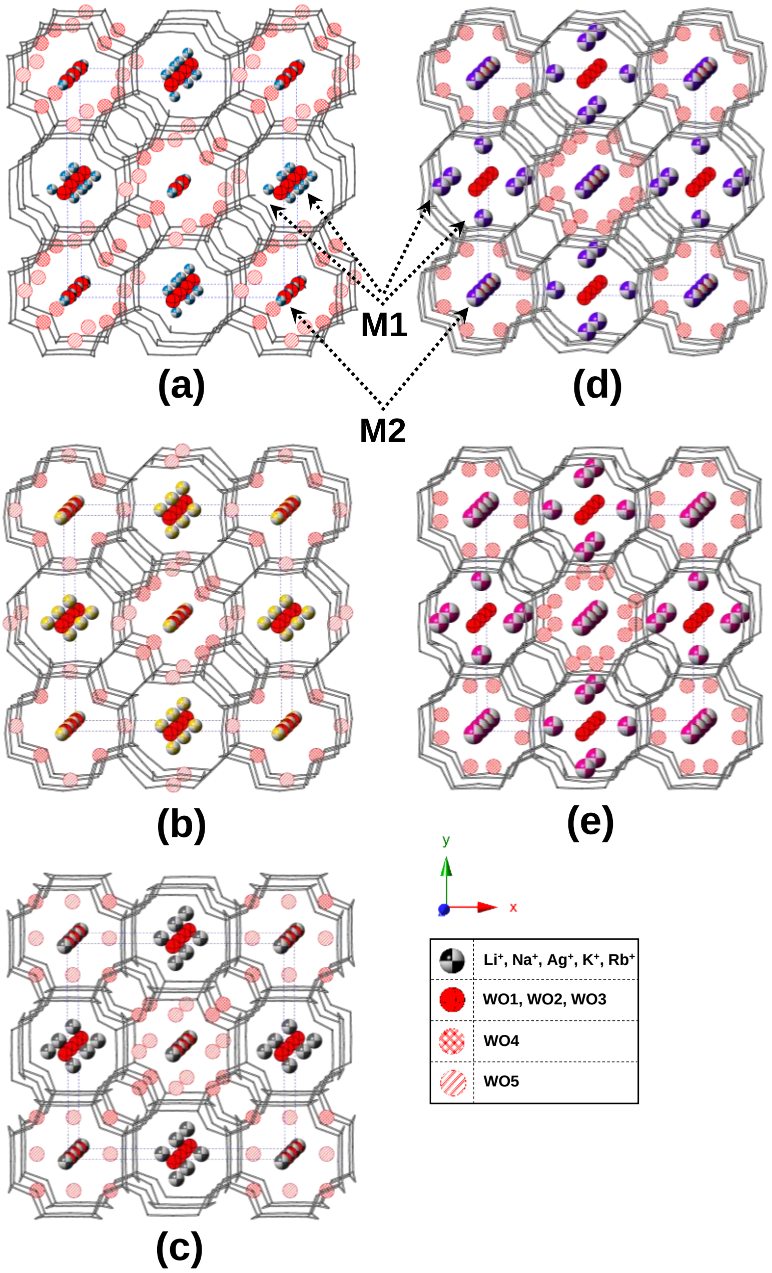

Figure S1. Polyhedral representations of (**a**) Li-MER, (**b**) Na-MER, (**c**) Ag-MER, (**d**) K-MER, and (**e**) Rb-MER along the (001) direction. Grey sticks represent disordered Al/Si framework. Each colored beach ball represents an extra-framework cation. Equatorial, hatched, and striped red balls represent oxygens of WO(1)–WO(3), WO(4) and WO(5), respectively.

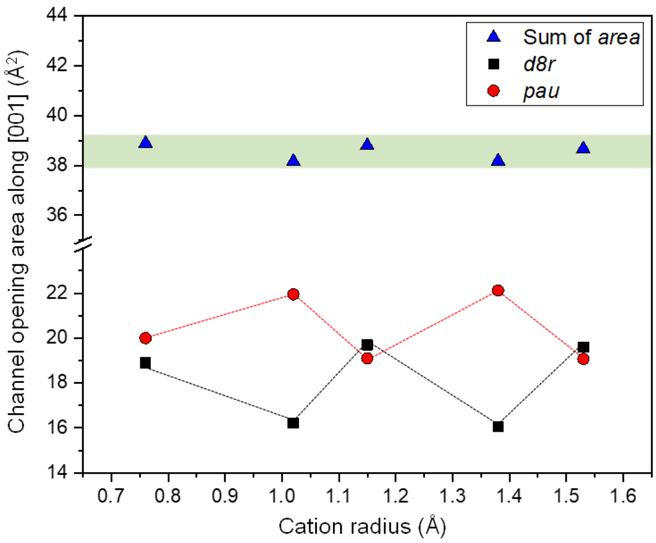

Figure S2. Comparison of channel opening area of *d8r* (black symbol) and *pau* (red symbol) along the (001) direction and sum of two areas.

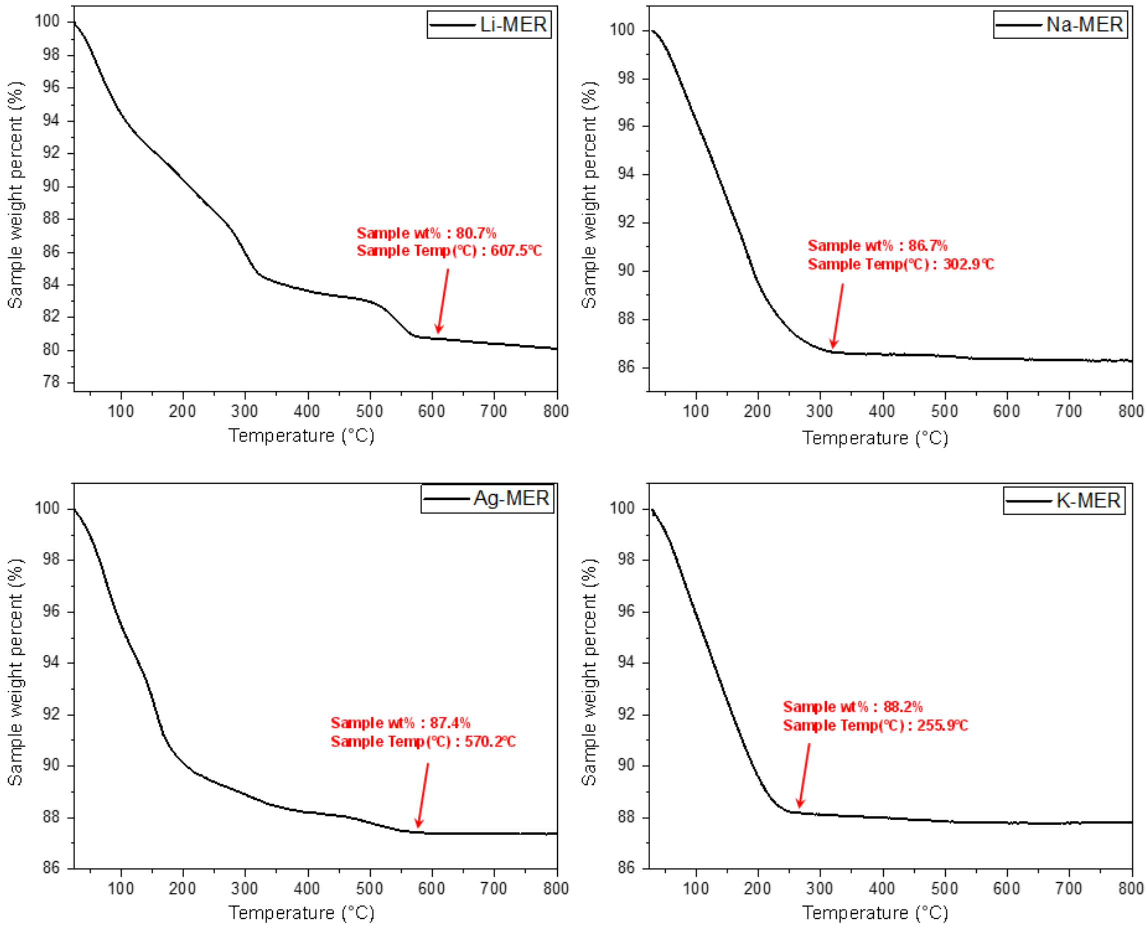

Figure S3. Thermogravimetric analysis results of Li-, Na-, Ag-, and K-MER.

Table S1. Cation-exchange conditions of MERs and chemical composition from Rietveld refinement and stoichiometric analysis.

Material	Reagent	Conc. Used (M)	No. of Treatments	Treatment Duration (days)	%H_2O[a]	Rietveld	EDX[b]
Li-MER	LiCl, (99%)	sat.	3	1	19.7	$Li_{6.9}Al_{6.9}Si_{25.0}O_{64.0}\cdot 26H_2O$	$Li_{6.8}Al_{6.8}Si_{25.2}O_{64}\cdot 26.0H_2O$
Na-MER	NaCl, (99%)	sat.	3	1	13.3	$Na_{7.5}Al_{7.0}Si_{25.0}O_{64.0}\cdot 20.0H_2O$	$Na_{7.5}Al_{7.0}Si_{25.0}O_{64}\cdot 17.8H_2O$
Ag-MER	AgCl, (99%)	sat.	3	1	11.9	$Ag_{7.0}Al_{7.0}Si_{25.0}O_{64.0}\cdot 22.2H_2O$	$Ag_{8.3}Al_{7.0}Si_{25.0}O_{64}\cdot 22.5H_2O$
K-MER[c]					11.8	$K_{6.4}Al_{6.5}Si_{25.8}O_{64.0}\cdot 15.3H_2O$	$K_{6.1}Al_{6.5}Si_{25.8}O_{64}\cdot 16.0H_2O$
Rb-MER[d]					9.82	$Rb_{6.4}Al_{6.3}Si_{25.7}O_{64.0}\cdot 14.9H_2O$	

[a] The water contents in wt%. Weight loss is measured by thermogravimetric analysis (TGA) up to ca. 800 °C; [b] Confirmed from chemical and energy-dispersive X-ray spectroscopy (EDS) and TG analysis; [c] Hydrothermal synthesis following method from Itabashi et al., 2008; [d] Results from Itabashi et al., 2008.

Table 2. Chemical composition calculated from energy-dispersive spectroscopy (EDS) method.

**Li-MER**						
Measurement		1	2	3		
Atomic percent (%)	Al	6.1(1)	6.2(1)	6.3(1)		
	Si	22.7(1)	23.0(1)	23.5(1)		
	K	0	0	0		
	O	71.1(1)	70.8(1)	70.2(1)		
**Na-MER**						
Measurement		1	2	3		
Atomic percent (%)	Al	5.7(1)	5.9(1)	5.4(1)		
	Si	20.4(1)	21.4(1)	19.3(1)		
	Na	6.2(1)	6.1(1)	6.0(1)		
	O	67.5(1)	66.4(1)	69.2(1)		
**Ag-MER**						
Measurement		1	2	3		
Atomic percent (%)	Al	5.3(1)	5.6(1)	5.4(1)		
	Si	18.6(1)	20.1(1)	19.4(1)		
	Ag	6.0(1)	6.7(1)	6.5(1)		
	O	70.1(1)	67.6(1)	68.6(1)		
**K-MER**						
Measurement		1	2	3	4	5
Atomic percent (%)	Al	6.48(5)	6.17(5)	6.25(5)	6.22(5)	6.26(5)
	Si	25.25(5)	25.49(5)	24.61(5)	25.00(5)	25.34(5)
	K	5.37(5)	5.39(5)	7.03(5)	6.31(5)	5.56(5)
	O	62.9(5)	62.94(5)	62.11(5)	62.48(5)	62.84(5)

Table 3. Refined cell parameters and atomic coordinates of M-MER at ambient conditions (M = Li^+, Na^+, Ag^+, K^+ and Rb^+) [a].

		**Li-MER**	**Na-MER**	**Ag-MER**	**K-MER**	**Rb-MER**[b]
Space group		*I4/mmm*	*I4/mmm*	*I4/mmm*	*I4/mmm*	*I4/mmm*
$_{w}R_{P}$(%), χ^2		1.91, 8.80	1.83, 0.36	2.50, 0.67	2.08, 3.01	
Chemical composition		$Li_{6.9}Al_{6.9}Si_{25.0}O_{64.0}\cdot26.0H_2O$	$Na_{7.5}Al_{7.0}Si_{25.0}O_{64.0}\cdot20.0H_2O$	$Ag_{7.0}Al_{7.0}Si_{25.0}O_{64.0}\cdot22.2H_2O$	$K_{6.42}Al_{6.5}Si_{25.8}O_{64.0}\cdot15.3H_2O$	$Rb_{6.4}Al_{6.3}Si_{25.7}O_{64.0}\cdot14.9H_2O$
Cell (Å)	a	14.1613(4)	14.1432(9)	14.1334(9)	14.1927(4)	14.1798(3)
	c	9.9493(4)	10.0275(7)	10.0403(10)	9.9560(5)	9.9308(2)
Cell volume (Å^3)	V	1995.25(14)	2005.81(25)	2005.56(28)	2005.46(15)	1996.76(7)
Si(1)	x	0.11125(19)	0.11030(7)	0.116571(27)	0.11051(10)	0.11046(14)
32o	y	0.25592(18)	0.25401(18)	0.25892(11)	0.25261(16)	0.25940(14)
	z	0.16212(18)	0.15681(18)	0.15376(13)	0.15966(15)	0.115637(14)
	Occu[c]	0.9815	0.9844	0.9844	0.9855	1.0
	U_{iso}[d]	0.0216(16)	0.013(4)	0.0140(24)	0.0232(14)	
O(1)	x	0.1194(6)	0.1189(5)	0.15375(22)	0.2823(5)	0.3046(4)
16l	y	0.2750(7)	0.2878(7)	0.2758(4)	0.1200(5)	0.1243(3)
	z	0.0	0.0	0.0	0.0	0.0
O(2)	x	0.0	0.0	0.0	0.0	0.0
16n	y	0.2509(6)	0.26579(27)	0.25700(20)	0.2657(4)	0.2510(6)
	z	0.2054(8)	0.2072(4)	0.1557(4)	0.2069(6)	0.1969(5)
O(3)	x	0.15347(32)	0.14234(21)	0.15702(12)	0.14141(22)	0.1561(4)
16m	y	0.15347(32)	0.14234(21)	0.15702(12)	0.14141(22)	0.1561(4)
	z	0.2089(8)	0.1697(10)	0.2096(6)	0.1778(7)	0.1991(5)
O(4)	x	0.1579(4)	0.18004(15)	0.15460(16)	0.18462(31)	0.1540(4)
16k	y	0.6579(4)	0.68004(15)	0.65460(16)	0.68463(31)	0.6540(4)
	z	0.25	0.25	0.25	0.25	0.25
M(1)	x	0.07(35)	0.0770(19)	0.0901(10)	0.1689(9)	0.592(2)
8j	y	0.5	0.5	0.5	0.5	0.1714(2)
	z	0.0	0.0	0.0	0.0	0.5
	Occu[c]	0.3625	0.4375	0.375	0.5	0.592(2)
	U_{iso}[d]	0.062(5)	0.042(9)	0.244(6)	0.038(5)	
M(2)	x	0.0	0.0	0.0	0.0	0.0
4e	y	0.0	0.0	0.0	0.0	0.0
	z	0.259(5)	0.2528(16)	0.2342(7)	0.2382(4)	0.2603(4)
	Occu[c]	1.0	1.0	1.0	0.606(11)	0.418(4)
WO(1)	x	0.0	0.0	0.0	0.0	0.0
2b	y	0.0	0.0	0.0	0.0	0.0
	z	0.5	0.5	0.5	0.5	0.5
	Occu	1.0	1.0	1.0	0.657(24)	1.0
WO(2)	x	0.0	0.0	0.0	0.0	0.0
2a	y	0.0	0.0	0.0	0.0	0.0
	z	0.0	0.0	0.0	0.0	0.0
	Occu	1.0	1.0	0.09(7)	1.0	1.0
WO(3)	x	0.0	0.0	0.0	0.0	0.0
4d	y	0.5	0.5	0.5	0.5	0.5
	z	0.25	0.25	0.25	0.25	0.25
	Occu	1.501(32)	0.44(4)	1.0	1.0	1.0
WO(4)	x	0.1461(6)	0.1365(15)	0.16964(13)	0.1840(12)	0.1859(9)
8h	y	0.1461(6)	0.1365(15)	0.16964(13)	0.0782(11)	0.0531(10)

16l [e]	z	0.5	0.5	0.5	0.5	0.5
	Occu	1.0	0.787(24)	1.0	0.5	0.430(4)
WO(5)	x	0.5	0.5	0.5		
8j	y	0.2705(8)	0.2490(19)	0.33022(13)		
	z	0.0	0.0	0.0		
	Occu	1.0	1.0	1.0		

[a] Esd's are in parentheses. WO denote oxygen site of H_2O molecules; [b] Model from Itabashi et al., 2008; [c] Occupancies were calculated by the result from EDS; [d] Isotropic displacement factors (Uiso) were refined by grouping the framework atoms and the extra-framework species, respectively; [e] Wyckoff position of 16l belongs to K- and Rb-MER.

Table 4. Selected interatomic distances (Å) and angles (°) for M-MER at ambient conditions (M = Li^+, Na^+, Ag^+, K^+, and Rb^+)[a].

	**Li-MER**	**Na-MER**	**Ag-MER**	**K-MER**	**Rb-MER**[b]
Si–O(1)[c]	1.6395(12)	1.64813(29)	1.6481(4)	1.6502(7)	1.330
Si–O(2)[c]	1.6349(13)	1.64815(28)	1.6479(4)	1.6480(7)	1.946
Si–O(3)[c]	1.6368(13)	1.64813(28)	1.6479(4)	1.6479(8)	1.823
Si–O(4)[c]	1.6402(13)	1.64806(27)	1.6482(4)	1.6458(7)	1.916
Mean[d]	1.6378(1)	1.64811(1)	1.6480(1)	1.6479(1)	1.753(1)
Si–O(1)–Si	159.4(6)	145.1(4)	139.02(25)	148.8(4)	119.456
Si–O(2)–Si	149.0(6)	142.35(23)	177.68(21)	144.25(31)	124.978
Si–O(3)–Si	124.5(5)	121.39(34)	119.38(15)	119.85(32)	109.975
Si–O(4)–Si	152.1(6)	177.36(27)	145.83(18)	172.1(5)	153.949
Channel opening area of *pau* unit along (010) direction	18.68	17.62	21.91	18.03	16.68
M(1)–O(1)	3(1)	3.059(13)	3.295(6)	3.166(7)	2.850
M(1)–O(2)				3.062(6)	3.205
M(1)–WO(3)	2.7(18)	2.733(11)	2.815(6)		
M(1)–WO(4)				2.364(17)	2.59
M(1)–WO(5)					
M(2)–O(3)	3.114(8)	2.967(8)	3.1482(23)	2.901(5)	3.130
M(2)–WO(1)	2.39(5)	2.478(16)	2.668(7)	2.607(4)	2.380
M(2)–WO(2)	2.58(5)	2.535(16)	2.352(7)	2.371(4)	2.585
WO(1)–WO(4)		2.729(30)		2.837(16)	2.741
WO(1)–WO(5)			2.3995(18)		
WO(3)–O(4)			3.0902(31)		3.088
WO(4)–O(1)			2.613(4)		
WO(4)–O(3)			2.926(6)		
WO(4)–WO(4)					2.633
WO(4)–WO(5)	2.382(6)	2.520(17)	2.3976(18)		
Bond Valence sum					
M(1)	0.102	0.426	0.251	1.48	0.642
M(2)	0.179	0.515	0.511	1.429	1.736

[a] Esd's are in parentheses, and WO denotes oxygen site of water molecules; [b] Model from Itabashi et al., 2008; [c] Interatomic distance was restrained by Al/Si ratio from EDS result; [d] Standard deviations computed using $\sigma = 1/n[\sum_{i=1}^{n} \sigma_i{}^2]^{1/2}$.
